# Biologic variability of human foreskin fibroblasts in 2D and 3D culture: implications for a wound healing model

**DOI:** 10.1186/1756-0500-2-229

**Published:** 2009-11-18

**Authors:** Mark A Carlson, Amy K Prall, Jeremiah J Gums, Alex Lesiak, Valerie K Shostrom

**Affiliations:** 1 Department of Surgery, University of Nebraska Medical Center, Omaha, NE 68198-3280, USA; 2 Department of Biostatistics, University of Nebraska Medical Center, Omaha NE 68198-4375, USA; 3 Surgery 112, Omaha VA Medical Center, 4101 Woolworth Ave, Omaha NE 68105, USA

## Abstract

**Background:**

The fibroblast-populated 3D collagen matrix is a model of tissue and healing which has been used since the 1980's. It was hypothesized that anchorage disruption of the collagen matrix would produce p53-dependent apoptosis in the embedded fibroblasts, but results of hypothesis testing were variant.

**Findings:**

The response of p53 to anchorage disruption in 3D culture or to UV irradiation in 2D culture was influenced both by fibroblast strain and culture conditions. It also was determined that data scatter in a collagen matrix contraction assay was related to fibroblast strain and possibly to technical factors, such as cell culture technician and/or number of matrices utilized. Subsequent analysis suggested that phenotypic drift and/or inter-strain genetic variability may have been responsible for the data scatter. In addition, several technical factors were identified that may have contributed to the scatter.

**Conclusion:**

Experimentation with human foreskin fibroblasts in both 2D and 3D culture can produce variant data. The underlying cause of the data scatter appears to be partially due to the biologic variability of the fibroblast.

## Introduction

Fibroblast survival in the collagen matrix appears to be upregulated in matrices anchored to a rigid substratum; if the matrices are not rigidly anchored, then there is induction of fibroblast apoptosis [1-3]. A previous report suggested that p53 was upregulated in the fibroblast-populated collagen matrix after disruption of rigid anchorage [4]. It then was hypothesized that the upregulation of p53 after matrix detachment regulated the induction of apoptosis. Follow-up experiments on p53, however, revealed a persistent and unsettling amount of data scatter. Secondary to the difficulty in producing consistent results, a correction to the initial report was published [5]. More studies subsequently were performed to investigate the cause(s) of data variability in experiments with the fibroblast-populated 3D collagen matrix. The conclusions drawn from these studies may be relevant to the planning of experimentation with this model, and to the interpretation of data derived thereof.

## Materials and methods

Fibroblast culture, the collagen matrix model, the TUNEL assay, BrdU labeling, quantitative immunofluorescence, cellular assays, immunoblotting, and other assays were utilized as previously described [2,4,6,7]; see Additional File 1 for details.

## Results

### p53 response in detached 3D collagen matrices

**Figure 1:**
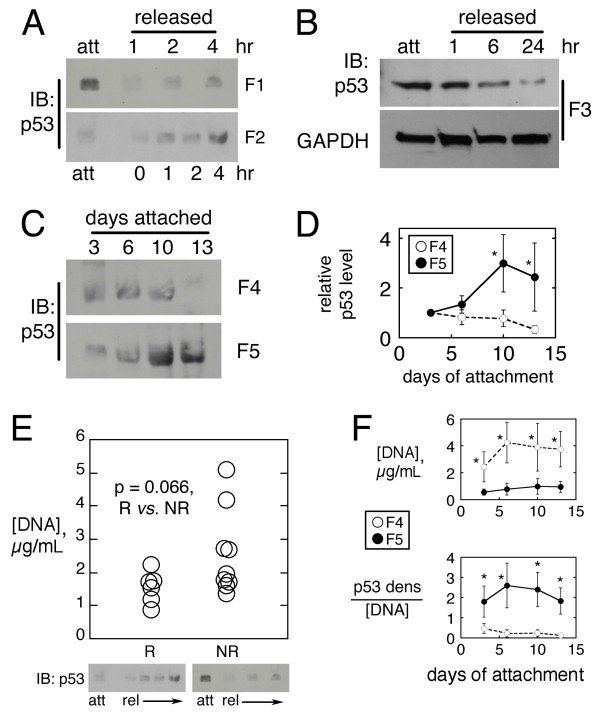
**p53 and DNA concentration in the fibroblast-populated collagen matrix**. (A) Immunoblot of p53 in attached *vs*. released matrices at indicated time post-release in two strains (F1 & F2; loaded with equal cell number; F1 data reproduced from previous work [4]). (B) Immunoblot of p53 and GAPDH in attached *vs*. released matrices for strain F3 (loaded with equal cell number). (C) Immunoblot of p53 in attached matrices for strains F4 *vs*. F5 over 2 weeks (loaded with equal cell number). (D) Densitometry of p53 bands in panel C (mean ± sd of 3 experiments). (E) Lysate [DNA] of attached matrices from responder (R; n = 6) *vs*. nonresponder (NR; n = 9) strains, with sample p53 immunoblot under each plot. Correlation coefficient shown is from nonparametric Wilcoxon testing. (F) Lysate [DNA] for data in panel D (upper), and p53 signal normalized to lysate [DNA] (lower); mean ± sd of 3 experiments. *p < 0.05, ANOVA.

Two different fibroblast strains at identical passage and culture conditions in the 3D collagen matrix demonstrated different p53 response to matrix release (Figure 1A). Strain F1 had a relatively high p53 level in the attached matrix, with rapid downregulation after release. Strain F2, however, had an opposite response--lower p53 level in the attached state, with induction after release. The level of p21 (a p53 transcriptional target) usually changed in step with p53 (data not shown). Another strain (F3) had a high p53 level in the attached state, with gradual decrease after matrix release (Figure 1B). In long-term experiments with attached matrices, the p53 level in strains F4 and F5 diverged (Figure 1C; densitometry in Figure 1D). In some fibroblast strains (not shown), the p53 level remained constant regardless of the state of matrix anchorage. Since culture conditions were held constant, it was suspected that the p53 response was dependent on the strain of cells (each strain was derived from a unique donor).

### p53 and DNA concentration in the 3D collagen matrix

Assay of DNA concentration may be used to quantify cell number in lysates of the fibroblast-populated collagen matrix [7]. Matrices prepared with different fibroblast strains and then cultured under identical conditions yielded lysates with divergent DNA concentrations (Figure 1E). The experiment shown in Figure 1A was performed on a total of 15 fibroblast strains (F1-F15), and each strain was classified on whether post-release p53 induction occurred (the responder group) or did not (the nonresponder group). The response status then was plotted against the DNA concentration of the attached matrix lysate (Figure 1E). There appeared to be a trend towards a higher lysate DNA concentration in the nonresponder group, which suggested that the cell population density of a nonresponder matrix tended to be higher than that in a responder matrix. This in turn suggested a relationship between fibroblast population density in the collagen matrix and the p53 response to matrix release.

### Dependence of population density and p53 on fibroblast strain

Lysate DNA concentration (attached matrix only) of strains F4 and F5 was plotted over a two week incubation period (Figure 1F, upper panel). By the day three, the DNA concentration of the F4 matrix was about five-fold greater than that in the F5 matrix. This implied that the generation times of the F4 and F5 strains differed. Densitometry then was performed on the p53 immunoblots of these strains, normalizing to lysate [DNA] (Figure 1F, lower panel). The F5 matrices (low population density) had a high p53 signal with respect to [DNA], while the F4 matrices (high fibroblast population density) had a low p53 signal with respect to [DNA]. That is, the more sparsely-populated F5 matrix expressed more p53 per cell than the more densely-populated F4 matrix. These two strains demonstrated different proliferative behavior and amount of p53 per cell in the attached collagen matrix. Furthermore, there appeared to be an inverse association between fibroblast population density and relative p53 expression in the attached state; that is, the more densely populated the matrix, the less the p53 detected per cell. Of note, cellular destruction was not a significant factor in these studies (see Additional File 1: Figure S1).

### Strain-dependent p53 response after UV exposure in 2D culture

**Figure 2:**
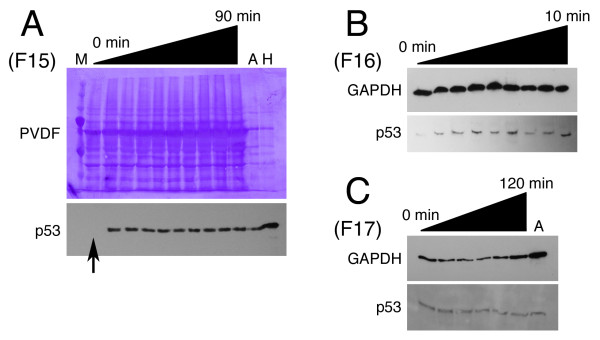
**Fibroblast variability in response to UV radiation and in a contraction assay**. (A-C) p53 immunoblot of monolayer fibroblasts (500,000 per 35 mm plate) immediately after a timed exposure (0-120 min) to the same UV source for 3 strains (F15-17). Coomassie-stained PVDF or GAPDH blot shown as a loading control. Arrow in panel A indicates lane of the nonradiated cells. M = molecular weight marker; A = A-431 control lysate; H = HeLa control lysate.

The response of strains F15-17 to UV radiation while in 2D (monolayer) culture is shown in Figure 2A-C. Strains F15 and F16 demonstrated induction of p53 after several minutes of UV exposure (Figure 2A-B). In contrast, strain F17 demonstrated no p53 induction with up to 2 hr of UV exposure (Figure 2C). So using a different stimulus (UV radiation), the p53 response in human foreskin fibroblasts again demonstrated inter-strain variability. Incidentally, the amount of fibroblast lysate (25 μg per lane) which yielded an adequate band in the p53 blots was about 10 times greater than that for the control lysates (A-431 or HeLa cells). This is evident in the Coomassie-stained membrane of the p53 immunoblot for strain F15 (Figure 2A). In order to avoid an excessive GAPDH band, only 5 μg of fibroblast lysate was loaded per lane for the these blots.

### Variability associated with a matrix contraction assay

**Figure 3:**
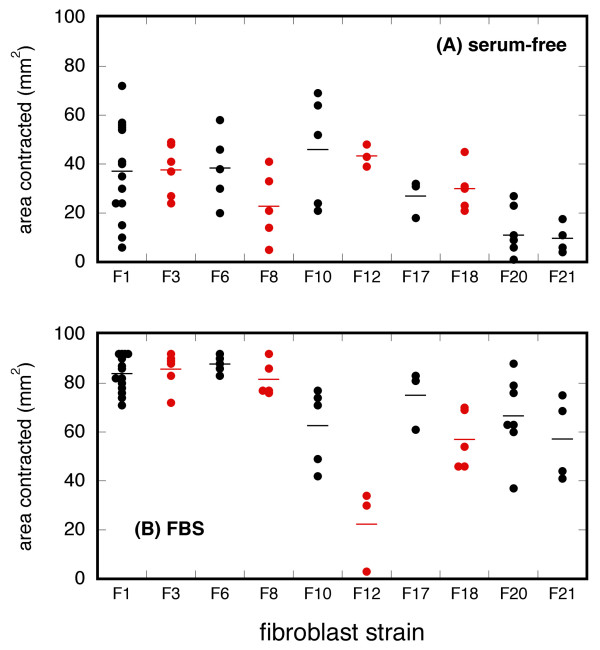
**Contraction of the fibroblast-populated collagen matrix in serum-free (A) or FBS-supplemented medium (B) in ten different fibroblast strains**. The strains denoted in this Figure are different from those in Figures 1 and 2. Each data point represents a unique experiment (mean of triplicate matrix areas); total experiments = 57. Each strain had 3-14 repeats (limited by strain availability). The horizontal line associated with each data column (strain) is the mean of all triplicate means for that strain (see Table S2 in Additional File 1 for the mean and standard deviation values).

Strain-dependent experimental variability also was observed with a floating collagen matrix contraction assay [8]. The results of this contraction assay on 10 fibroblast strains in DMEM with or without FBS is shown in Figure 3. A large amount of intra-strain (i.e., within column) variability was present, as evident by contraction standard deviations which ranged from 10-95% of the value of the mean (Additional File 1: Table S2) with serum-free media. Even strain F1 had a standard deviation that was 54% of the mean after 14 repeats. The contraction results of Figure 3 also demonstrated a large amount of inter-strain (i.e., among columns) variability; the contraction means in serum-free or FBS-supplemented medium ranged from 9.7-46.0 or 22.3-87.8 mm^2^, respectively, representing four-fold ranges of experimental results. Simultaneous and identical contraction assays performed by different technicians yielded similar results (data not shown), indicating that technician variability during the actual contraction assay probably did not contribute to the data scatter. The same lot of FBS was used for all contraction assays, so variability in serum-stimulated contraction presumably was not secondary to differences in serum activity.

Various assay conditions (cell strain, passage, etc.; see Additional File 1: section S9.) were recorded during the contraction experiments. In an attempt to determine whether one or more of these conditions were associated with contraction (possibly providing an explanation for the variability), statistical testing for associations was undertaken. Univariate analysis of recorded assay conditions revealed that (1) contraction in both serum-free and FBS-supplemented medium was associated with cell strain; (2) contraction in serum-free medium was associated with technician performing monolayer culture; and (3) contraction in FBS-supplemented medium was associated with number of matrices set up (all p < 0.05). Multivariate analysis confirmed the association of both contraction types with cell strain (p < 0.05), but no other significant associations were found. Details of the statistical analysis are in Additional File 1.

### Effect of cell culture conditions on p53

**Figure 4:**
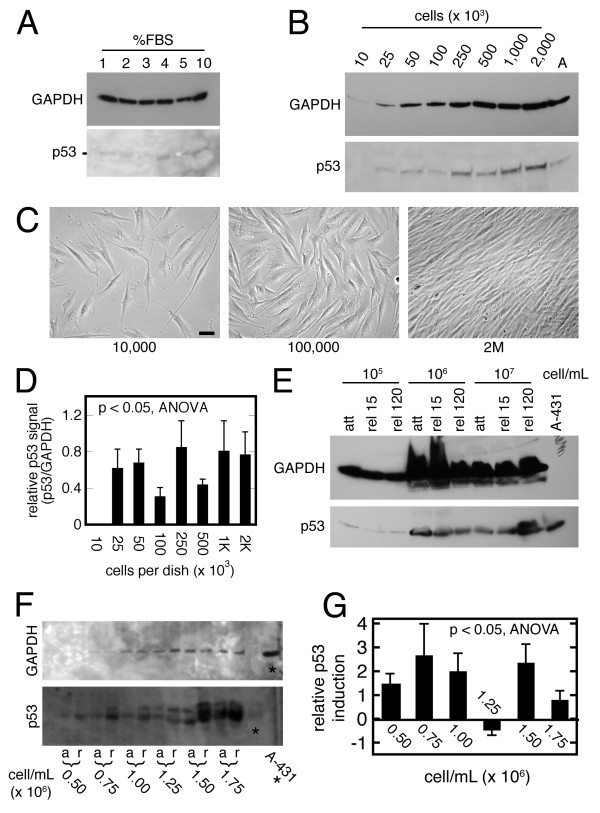
**Effect of culture conditions on p53 in human foreskin fibroblasts**. (A) Monolayer cells (50,000/35 mm dish) were cultured for 24 hr in FBS as indicated, followed by immunoblotting for p53 and GAPDH (loaded with equal cell number). The image of the p53 immunoblot has been contrast-enhanced. (B) Monolayer cells at the indicated plating density (35 mm dish) were grown for 24 hr, followed by immunoblotting for p53 and GAPDH (equal volumes); A = A-431 control lysate. (C) Phase images from panel B; bar = 20 μm. (D) Relative p53 densitometry from 3 experiments of the type in panel B. (E) Attached (att) collagen matrices containing fibroblasts at the indicated starting concentration were incubated for 24 hr prior to being released (rel) for the indicated minutes, followed by immunoblotting for p53 and GAPDH (loaded with equal cell number); control lysate A-431 on p53 gel only. (F) Similar experiment to panel E, except over a narrower cell concentration range; a = attached; r = released. (G) Relative p53 densitometry from 3 experiments of the type in panel F.

Effects of various culture conditions on p53 immunoblotting were tested for a contribution to experimental variability (Figure 4). Monolayer fibroblasts incubated in 1-10% FBS expressed different levels of p53; the highest relative expression occurred at 4% FBS (Figure 4A). Monolayer fibroblasts then were plated at different densities (Figure 4C) and then immunoblotted for p53 and GAPDH (Figure 4B). The relative p53 signal (Figure 4D) varied over a 4-fold range. In 3D culture, collagen matrices with 10^5^-10^7 ^cell per mL (starting population density) underwent a 2-hr period of release, and then were immunoblotted for p53 and GAPDH (Figure 4E). In this particular strain, there was no induction of p53 after matrix release at cell concentrations of 10^5 ^or 10^6 ^per mL (for reference, 10^6 ^cell per mL was the usual starting density elsewhere in this report). At 10^7 ^cells per mL, however, there was strong p53 induction at 120 min after matrix release. This experiment was repeated in a different fibroblast strain over a narrower cell concentration range (0.5-1.75 × 10^6 ^per mL; Figure 4F). Densitometry demonstrated p53 induction after matrix release at all but one cell concentration (Figure 4G). The magnitude of this induction, however, varied three-fold over the cell concentration range. The results of Figure 4 suggested that differences in cell culture conditions could influence the response of p53 to disruption of matrix anchorage, independent of strain effect. The effect of pH, temperature, and CO_2 _concentration was not evaluated.

## Discussion

Experiments on p53 biology in 2D or 3D culture models with human foreskin fibroblasts produced data scatter. There were at least four possible causes/explanations for this data scatter: (1) technical factors; (2) model reliability; (3) inter-strain diversity; and (4) intra-strain phenotypic drift. The identity of the technician performing the monolayer culture or the number of matrices utilized in a given experiment may have influenced the amount of matrix contraction (Figure 3 and statistical analysis in Additional File 1). The technician and cell strain variables were confounded in this analysis (particularly with serum-free medium), however, so a firm conclusion regarding the technician variable was not possible. Subtle variations in matrix culture conditions also may have contributed to experimental variability (Figures 4).

With regard to model reliability, the preponderance of the published data since the 1970's suggests that the 3D collagen matrix is a reliable model. The authors have no comparative reliability data on 2D *vs*. 3D culture. The absence of prior publications which report data scatter in this model might be secondary to publication bias for statistically significant data [9]. Nonsignificant or inconsistent data, while potentially important, may be subject to a negative publication bias (i.e., either are not submitted or not accepted for publication).

The data in this report may have been variable because the biology was variable. This supposition is supported by the inter-strain variability seen in all four Figures, and the fact that the statistical analysis determined that matrix contraction was dependent on fibroblast strain. Identical experiments performed on different fibroblast strains yielded dissimilar results. This suggests that subtle genetic and/or epigenetic differences might have been present among fibroblast strains (genomic analysis was not performed). If the data scatter had only been present between one strain and another, then inter-strain diversity might have been a complete explanation. Intra-strain variability (that is, dissimilar results from identical experiments performed on the same strain) also was observed (Figure 3). The cause for this intra-strain imprecision is not clear from this report; perhaps the phenotype of the human foreskin fibroblast may be more fluid than previously appreciated.

Some cell lines may not maintain a constant phenotype in tissue culture [10-12]. This phenomenon has been known as "phenotypic drift" [13,14]. It has not been extensively studied per se in human foreskin fibroblasts, although dermal fibroblasts have been shown to evolve phenotype both *in vivo *and in culture [15,16]. In primary cells, one explanation for phenotypic drift might be the onset of replicative senescence after prolonged passaging [17]. Low passage (= P9) neonatal fibroblasts were utilized in the present report, so replicative senescence probably was not a factor.

Regarding regulation of p53 level in 2D and 3D fibroblast culture, there was a tendency for rapidly-growing cell strains to express relatively less p53 in the collagen matrix when the initial cell concentration was 10^6 ^per mL, while slower-growing strains had greater p53 induction with loss of matrix anchorage (Figure 1). Seeding the cells at 10^7 ^cell per mL in some strains precipitated p53 induction after matrix release, which was not seen at lower population densities (Figure 4). Figure 1 and Figure 4 may appear to contradict, in that matrices with high DNA concentration in Figure 1*vs*. 4 had low *vs*. high p53 expression, respectively. This inconsistency might be reconciled by the fact that the initial matrix cell concentration in Figure 1 was 10^6 ^cell per ml in Figure 1, while it was 10^7 ^per mL in Figure 4 (panel E). It is likely (though not proven experimentally) that the fibroblast strain in 4E was "slow growing," so at a matrix concentration of 10^7 ^per mL (i.e., forced overcrowding), the strain upregulated p53. The "rapid growing" strains presumably could tolerate more overcrowding with less p53 expression, so they could attain a higher [DNA] concentration before upregulating p53. These observations are consistent with known p53 biology in other systems [18]. With regard to the relative p53 levels in 2D *vs*. 3D culture, there is not enough data to make a conclusion.

It has not been uncommon to focus on a single strain of cells in experimentation with the 3D collagen matrix. Such practice may decrease variability arising from putative inter-strain diversity; however, if the chosen strain is aberrant, then the investigator may produce precise but inaccurate data. Conversely, while the use of multiple fibroblast strains may decrease experimental precision, such practice should minimize the risk of sampling error. In conclusion, experimentation with one strain and/or with few experimental repeats may be misleading. Although not proven in this report, it was suspected that both inter-strain diversity and intra-strain phenotypic drift were present. Evidence for these phenomena would require extensive molecular investigation which has not yet been accomplished. Regarding experimentation with human neonatal foreskin fibroblasts, the main messages of this report are: (1) give consideration to using multiple strains; and (2) data scatter requires more experimental repeats. Currently there is inadequate data to establish the precise number of strains and repeats. Possible technical contributions to data scatter, as suggested by Figure 3, emphasize the need for maintaining standardization of culture and assay conditions.

## Abbreviations

2D, 3D: two or three dimensional; ANOVA: analysis of variance; DMEM: Dulbecco's Modified Eagle Medium; EDTA: ethylenediaminetetraacetic acid; DMSO: dimethyl sulfoxide; FBS: fetal bovine serum; GAPDH: glyceraldehyde 3-phosphate dehydrogenase; HEPES: 4-(2-hydroxyethyl)-1-piperazineethanesulfonic acid; PBS: phosphate buffered saline; PVDF: polyvinylidene difluoride; SDS-PAGE: sodium dodecyl sulfate polyacrylamide gel electrophoresis; SF: serum-free; UV: ultraviolet; WST-1: 4- [3-(4-Iodophenyl)-2-(4-nitrophenyl)-2H-5-tetrazolio]-1,3-benzene disulfonate.

## Competing interests

The authors declare that they have no competing interests.

## Authors' contributions

MAC designed the experiments, supervised the research, and directed the manuscript writing. AKP, JJG, and AL assisted in experimental design and performed the experiments. VKS performed statistical analysis. All authors read and approved the final manuscript.

## Supplementary Material

Additional file 1 **Supplemental Material**. Materials and Methods; Figures S1-2; supplemental material for Figure 3, Tables S1-S4. Click here for file
