# Supplementary material for: Biologic variability of human foreskin fibroblasts in 2D and 3D culture: implications for a wound healing model
Source: BMC Res Notes. 2009 Nov 18;2:229. doi: 10.1186/1756-0500-2-229 (PMC2784473; doi:10.1186/1756-0500-2-229)
Supplement: Additional file 1 — Supplemental Material. Materials and Methods; Figures S1-2; supplemental material for Figure 3, Tables S1-S4. [file 1756-0500-2-229-S1.PDF]

Carlson et al.

Biologic variability of human foreskin fibroblasts in 2D and 3D culture: implications for a wound healing model

Supplemental Material

Table of Contents

Materials and Methods	2-9
Figure S1	10-11
Figure S2	12-13
Supplemental Material for Figure 3	14-15
Table S1	16
Table S2	17
Table S3	18
Table S4	19
References for Supplemental Material	20

Supplemental Material: Materials And Methods

(Step-by-step protocols on all methods are available from corresponding author)

S1. Isolation of fibroblast strains from neonatal foreskin

The use of primary dermal fibroblasts derived from human donors without subject identifiers was approved by our Institutional Review Board. Fibroblasts were cultured from explants of human neonatal foreskin as previously described [1, 2]. Each cell strain was derived from a single donor. Since donor tissue was foreskin, all donors were male infants. As stated above, data on donor ethnicity, obstetrical history, maternal medical history, etc. was not recorded per IRB protocol. Each foreskin was placed into iced DMEM (Invitrogen™ Cat. No. 12800-017, with 44 mM $NaHCO_3$ and 20 mM HEPES added, pH 7.2) supplemented with antibiotic/antimycotic solution (Invitrogen™ Cat. No. 15240-062, used per manufacturer recommendation) immediately after circumcision and stored briefly at 4˚C.

Within 24 hr after collection, dermis was dissected from the specimen, minced, and placed into a 100 mm culture plate. The dermal explants were covered with 20 mL of growth medium, which was DMEM supplemented with 10% FBS, antibiotic/antimycotic solution as described in the preceding paragraph, and supplemental amphotericin B (Fungizone®, Invitrogen™ Cat. No. 15290-018, final concentration 6 µg/mL). Growth medium was exchanged every 3-4 days. Once the plate was 80-90% confluent (about 2-3 weeks later), the cells were trypsinized and transferred to a T75 flask. The cells were passaged to P3 by trypsinizing at 80-90% confluency (see Figure S2). Subconfluent cells at P3 were placed in cryopreservative (9:1 FBS:DMSO) at a concentration of 2-3 million cell per mL, and then cooled down slowly (over 24 hr) to -80˚C before being put into liquid nitrogen for long-term storage.

S2. Expansion of each cell strain

In order to generate sufficient flasks of cells for repeat experiments, a vial of frozen P3 fibroblasts was rapidly thawed in a 37˚C water bath and plated into a T75 flask with 20 mL of growth medium. Fibroblast strains utilized in the experiments of this manuscript were expanded with different batches of FBS; records specifying which batch was used for each strain were not kept. Cells were passaged as above to P7, at which point there would be 32 flasks (refer to the flow diagram in Figure S2). These flasks of P7 then were frozen down as described in section S1. A cryovial of P7 fibroblasts could be thawed as needed, plated, and then grown out to P9, at which point experiments with monolayer cells or collagen matrices could be performed. Experiments in this report were performed at P9, with the exception of Figure 3, in which a range of cell passages was tested.

S3. Monolayer experiments with UV irradiation

Fibroblasts at P9 were trypsinized, plated into 35 mm round culture dishes (250,000 per plate), and covered with 4 mL of growth medium. Cells were incubated at 37˚C for 48 hr prior to UV irradiation, which was done by placing the uncovered plates at a distance of 72 cm from the ultraviolet lamp (Sylvania, No. G30T8) in a class II, Type A2 Biological Safety Cabinet (NuAire Labgard NU-425-300) for the times specified in Figure 2. This 30 watt germicidal lamp supplied 85 microwatts per cm^2 at a distance of 100 cm from the source [3].

S4. Fibroblast-populated collagen matrix

The collagen matrix model was utilized as previously described [1, 2, 4, 5]. Fibroblasts at P9 were trypsinized from T75 flasks, resuspended in DMEM, and underwent quadruplicate hemacytometer counting prior to being processed into collagen matrices. Fibroblasts (final concentration = 10^6 per mL) were mixed with solubilized type I bovine collagen (PureCol™, Advanced BioMatrix; final concentration = 1.5 mg/mL) in DMEM (final pH 7.2). This solution was incubated at 37˚C with continuous swirling for 4 min prior to pipeting a 200 µL aliquot (containing 2×10^5 cells) into each well (containing a 12 mm circular inscription) of a 24-well culture plate. The 200 µL aliquot would form a bead within the inscription. The plate then was incubated for 1 hr @ 37˚C, during which time each aliquot would polymerize to form a fibroblast-populated collagen matrix. Two mL of growth medium (supplemented with 50 µg/mL of ascorbate) was added to each well after polymerization. For experiments utilizing attached matrices (Figures 1, 4, and Figure S1), the growth medium was exchanged after 72 hr, and the experiment would begin. Matrix release (detachment) was performed by gently lifting it off the culture well with a smooth, rounded metal spatula.

S5. Cell retrieval from the collagen matrix

The cell counting in Figure S1 required retrieval of fibroblasts from the collagen matrix [2]. A matrix was washed twice with PBS and then incubated with 100 µL of 0.025% trypsin/0.01% EDTA in PBS (Invitrogen™, Cat. No. R-001-100) for 10 min at 37°C. A 400 µL aliquot of 5 mg/mL collagenase (Type 1A, Sigma) in 130 mM NaCl, 10 mM Ca acetate, 20 mM HEPES (pH 7.2) was directly added to each well, and then the plate was placed on a rotary agitator (250 rpm) for 30–45 min at 37°C. The enzymatic digestion was quenched with 50 µL of

FBS, the cells were pelleted with a 4 min centrifugation at 600*g*, and the cell pellet was resuspended in 200 μL of DMEM for subsequent counting with a hemacytometer.

S6. DNA assay

The DNA assay was adopted from a standard protocol [6]. Lysates were clarified with centrifugation (20,000*g*, 30 min, 4˚C), and then supernatant (10 μL) was added to 5 ng/mL bisbenzimide (Hoechst 33258; Sigma) in PBS (final volume 2 mL; performed in triplicate). Fluorescence was read on a spectrophotometer (Perkin Elmer L550B) using excitation/emission settings of 356/458 nm; sample concentration was determined against DNA standards of 0-1 μg/mL.

S7. WST-1 assay

The WST-1 assay (Cell Proliferation Reagent WST-1; Roche Applied Science) was performed as previously described [4]. WST-1 reagent (20 μL) was added to each well, which contained 1.0 mL of growth medium and a single fibroblast-populated collagen matrix, as described in section S4. After a 2 hr incubation, absorbance (420-480 nm) of the medium was read on a Perkin Elmer L550B spectrophotometer.

S8. Immunoblotting and densitometry

These procedures were performed as previously described [2]. At the indicated time points post-release, matrices were placed on ice and washed three times with ice-cold PBS, and dounced (50 strokes) in ice-cold lysis buffer (500 μL per 18 matrices) containing 50 mM Tris (pH 7.4; Sigma), 150 mM NaCl, 1% NP40 (Sigma), 1% sodium deoxycholate (Sigma), 0.1%

SDS (Sigma), 5 mM EDTA (Sigma), 50 mM sodium fluoride (Sigma), 0.1 mM sodium vanadate (Sigma), 1 mM AEBSF (Sigma), 1 µg/mL pepstatin A (Sigma), and 1 µg/mL leupeptin (Sigma). Crude lysates were incubated on ice for 30 min, and centrifuged at 25,000*g* for 30 min at 4˚C. A 50 µL aliquot of the supernatant was saved for the DNA assay, and the remainder was boiled for 4 min in reducing buffer. SDS-PAGE was performed on 7% polyacrylamide gels. Each immunoblot lane typically was loaded with 10 µg of cellular protein [4]. Protein was transferred to PVDF membrane (Immobilon-P, Millipore) and then blotted with antibodies to α-tubulin (Sigma), p53 (clone Ab-7, Calbiochem), GAPDH (ab9485, Abcam). The HeLa and A-431 control lysates were from Santa Cruz and Calbiochem, respectively. Densitometry was performed with a public domain image analysis program (ImageJ, http://rsb.info.nih.gov/ij/). For the purpose of analysis, a "responder" fibroblast strain was defined as that demonstrating induction of p53 (by immunoblotting) after matrix release; a "nonresponder" strain was defined as that demonstrating no induction (or downregulation) of p53 after matrix release. Relative p53 expression in this manuscript was quantified with densitometry of immunoblots. The lane loading of immunoblots was based on DNA content for all experiments in this manuscript. The DNA assay results were not shown in all Figure panels; in some cases, a GAPDH blot or Coomassie blot was shown as a visual demonstration of lane loading.

S9. Matrix contraction assay

Matrices were set up per section S4, and covered with 1.0 mL of DMEM with or without 5% FBS. Immediately after addition of medium, each matrix was detached from the well as described in section S4. The matrices were incubated for 4 hr at 37˚C, and then fixed with 10% buffered formaldehyde. The matrices were carefully mounted on a culture plate lid with a

millimeter reference, and scanned against a black background with a letter-sized flatbed scanner. Matrix area was measured from the digital image using ImageJ (see section S8). Matrix area for a given timepoint was the mean of triplicate matrices; the starting area (t = 0) was determined by fixing triplicate matrices immediately after release. The contracted area (as used in Figure 3) was defined as the starting (t = 0) area minus the final (t = 4 hr) area. For every contraction experiment performed, there was data collected on both serum-stimulated and serum-free contraction. FBS used for all serum-stimulated contraction assays was from the same batch, stored at -20˚C in aliquots.

Data on the following parameters were recorded for each matrix contraction experiment:

1. Matrix area at t = 0, in mm^2
2. Matrix area at t = 4 hr in DMEM (i.e., serum-free media), in mm^2
3. Matrix area at t = 4 hr in DMEM supplemented with 5% FBS, in mm^2
4. Cell strain (denoted as F1, F2, etc.)
5. Cell passage number at the time the monolayer fibroblasts were trypsinized to make collagen matrices, recorded as a whole number
6. Confluency of the monolayer fibroblasts at the time the cells were trypsinized to make collagen matrices, recorded as a percentage
7. Duration between day of last T75 flask splitting to day of T75 trypsinization to make collagen matrices (i.e., how long fibroblasts were in their final T75 passage before making matrices), recorded in days
8. Number of cells recovered from the T75 culture flask after trypsinization to make collagen matrices, recorded as cells per flask

9. Number of matrices set up, indicated by the total volume (in μL) of collagen utilized
10. Technician who cultured the cells in monolayer prior to the contraction experiment

With regard to parameter 10 (technician status), the technician (T1) who performed the actual contraction assay was the same person for all experiments shown in Figure 3; however, multiple technicians were involved in monolayer cell culture. So the technician-dependent variable in this analysis referred to the person who was culturing the fibroblasts for the contraction assay (not technician T1 who actually performed the contraction assay). As mentioned in section on matrix contraction in the manuscript, data from parallel contraction assays performed by a second technician as a control for technician T1 were not included in the body of contraction data that was analyzed. All of the above parameters were analyzed retrospectively; i.e., the parameters were not varied in a prospective, controlled manner.

S10. Technician responsible for performance of experiments

Clusters of experiments which constituted a Figure panel or set of related panels were performed by a single technician. For example, all the Experiments in Figure 1 were performed by the same technician.

S11. Statistics

Demonstrative experiments (e.g., immunoblots, DNA assays) were replicated a minimum of three times on each strain of fibroblasts unless otherwise indicated in the Figure legends.

Numerical data were expressed as the mean ± standard deviation. Unless otherwise indicated, statistical testing on unpaired data was performed with analysis of variance (ANOVA), using a level of significance of $p < 0.05$. For statistical analysis of Figure 3, see subsequent section.

Supplemental Material: Figure S1

Assays of Cell Quantity in the Fibroblast-Populated Collagen Matrix

Modulation of p53 after matrix release may have been secondary to cellular destruction associated with physical detachment. In order to determine if cellular destruction was a factor, four different assays of cellular quantity (immunoblotting, colorimetric conversion, [DNA], and cell counts [4]) were performed on attached and released matrices (Figure S1). Two identical groups of six matrices were set up, incubated for 72 hr, and then one group was released. After another 24 hr, three matrices from each group underwent the WST-1 assay, one matrix from each group underwent cell retrieval for quadruplicate hemacytometer counting, and lysate was made from the remaining two matrices in each group for immunoblotting and the DNA assay. The experiment was performed on three different strains (one experiment per strain). GAPDH and α-tubulin immunoblotting, cell counts, WST-1 absorbance, and lysate DNA concentration are shown in A-D, respectively. The cropped band in the immunoblots (A) is a monolayer control lane. None of these cell quantity assays indicated that detachment of the fibroblast-populated collagen matrix was associated with a significant loss of cell number ($p > 0.05$, ANOVA), so it is unlikely that cellular destruction contributed to variability in p53 determination.

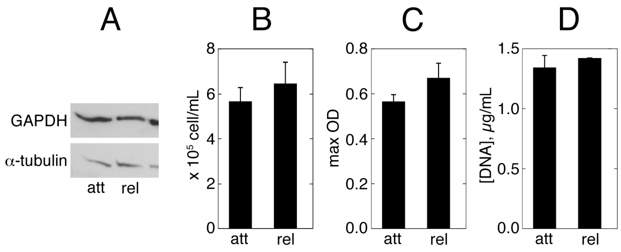

Carlson et al./Supplemental Figure 1

Supplemental Material: Figure S2

Protocol for culturing primary human fibroblasts from P3 to P9

Cryovials of P3 fibroblasts were prepared as described in section S1. A P3 cryovial was thawed, plated, and split according to the flow diagram in Figure S2. The weekdays of specific steps (labeled in red) are shown in the Tables embedded within the Figure. A passage was defined as the process of trypsinizing the cells and replating them, whether into 1, 2, or 4 flasks. Passaged cells were not split more than 1 to 4. Cells were counted every time they were trypsinized (i.e., at steps 2, 3, 4, 5, 7, and 8). Subconfluency was defined as ~90% of the dish being covered with cells, as viewed with inverted phase microscopy. After 32 flasks of P7 were obtained (step 5), cells were frozen as described in section S1. An experiment was performed by thawing a cryovial of P7, growing the cells up to P9, and then setting up the experiment with P9 cells.

schedule **(steps 1-6)**

week	Mon	Tues	Wed	Thu	Fri
1			thaw & plate		split 1:4
2	split 1:4		split 1:2		freeze

schedule **(steps 6-8)**

week	Mon	Tues	Wed	Thu	Fri
1			thaw & plate		split 1:4
2	make gels				

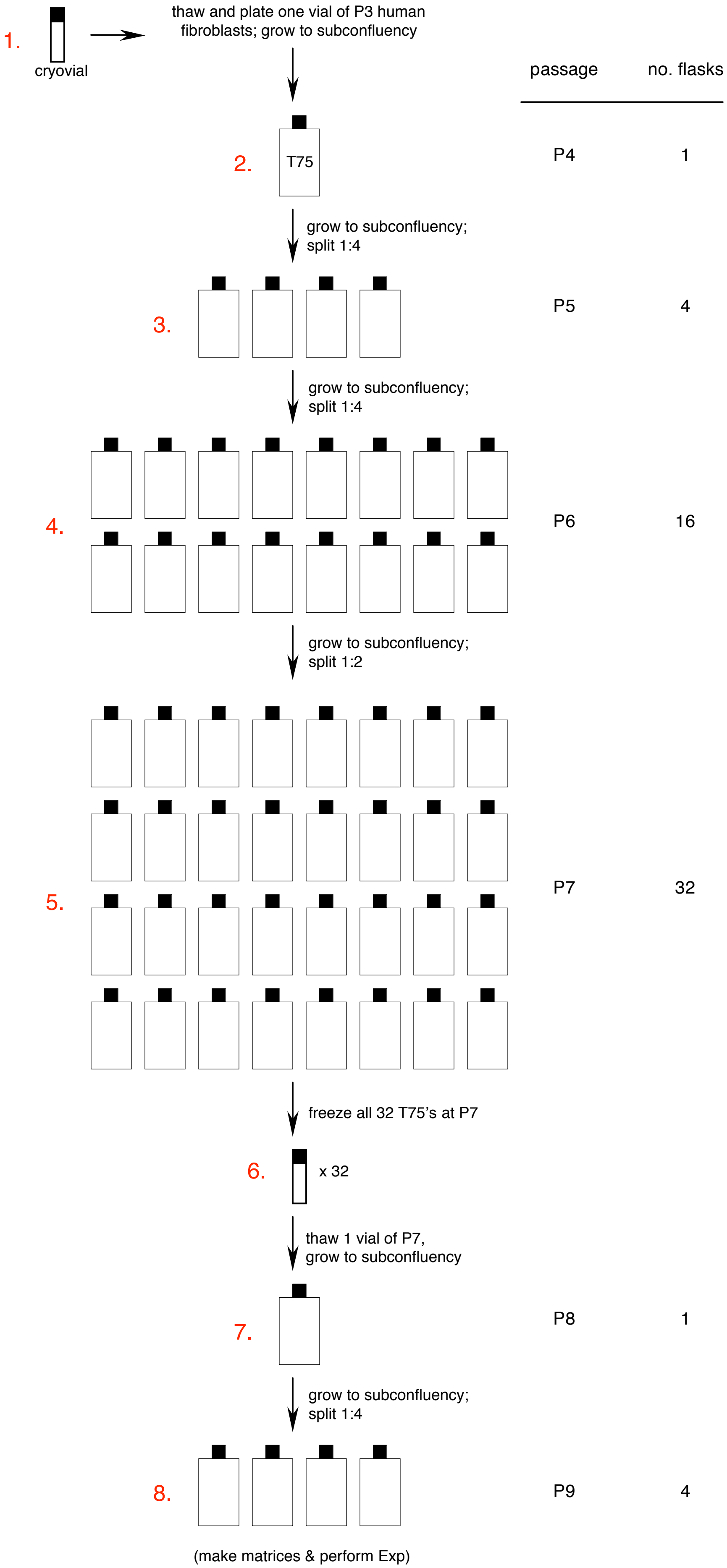

Carlson et al./Supplemental Figure 2

Supplemental Material for Figure 3

Introduction

The relationship of contraction of the fibroblast-populated collagen matrix in either serum free medium (DMEM) or FBS-supplemented medium with certain independent variables (described under section S9) was examined.

Statistical Methods

For Figure 3, PC SAS version 9.2 (http://www.sas.com/) was used for all analyses. Contraction with serum-free (SF) medium and FBS-supplemented medium was analyzed separately. Pearson correlation coefficients were calculated for each of the continuous variables (cell passage number, period of incubation prior to trypsinization, cell confluency just prior to trypsinization, cell number per flask at trypsinization, and volume of collagen utilized) with SF medium and FBS-supplemented medium. ANOVA was used to examine the relationship between the categorical variables (cell strain, technician who cultured cells) and contraction in SF or FBS-supplemented medium. From the univariate analyses (the Pearson correlations and ANOVAs), those that resulted in p-values less than 0.20 were put into a model together and examined jointly. From this point on, the level of significance was set to be 0.05. The term in the joint model with the largest nonsignificant p-value was dropped, and this process was repeated until all terms in the model were statistically significant. Both cell strain and technician were examined separately for their association with contraction in SF medium or FBS-supplemented medium. However, cell strain and technician were confounded, so only cell strain was used in the multivariate model. Tukey's adjustment for multiple comparisons was used on the final

model comparisons. A comparison of contraction in matrices treated with serum-supplemented *vs.* serum-free medium was not performed.

Results

A spreadsheet showing all the raw data of this analysis is available from the first author (MAC). The results from the univariate analyses are shown in Table S1. For contraction in SF medium, only cell strain had a p-value < 0.20. In FBS-supplemented medium, variables with $p < 0.20$ included cell strain, passage number, cell/flask, and collagen volume. All four of these variables were included in a multiple regression model for contraction in FBS-supplemented medium. After the model simplification process described in the methods section was applied, only cell strain remained in the model. The means and standard deviations of contraction for each cell strain in SF or FBS-supplemented medium are shown in Table S2. The Tukey adjusted p-values for all pairwise comparisons of contraction in SF medium between cell strains are given in Table S3; the analogous p-values for contraction in FBS-supplemented medium are given in Table S4.

Discussion

See main body of manuscript.

Table S1. p-values from univariate analyses.

Variable	SF medium	FBS medium
Passage No.	0.32	0.07
Incubation Period	0.28	0.76
Confluency	0.57	0.33
Cell/Flask	0.40	0.06
Collagen Volume	0.73	0.01
Cell Strain	< 0.01	< 0.01
Technician*	< 0.01	0.17

*Not included in multivariate analyses since confounded with cell strain

Table S2. Collagen matrix contraction in serum-free or FBS-supplemented medium.

		serum-free			FBS-supplemented		
Cell strain	Repeats	mean	sd	sd as % of mean	mean	sd	sd as % of mean
F1	14	37.1	20.0	54	83.9	7.3	9
F3	6	37.7	10.5	28	85.7	7.3	9
F6	5	38.4	14.6	38	87.8	3.5	4
F8	5	22.8	14.4	63	81.6	7.1	9
F10	5	46.0	22.3	48	62.6	15.9	25
F12	3	43.3	4.5	10	22.3	16.9	76
F17	3	27.0	7.8	29	75.0	12.2	16
F18	5	30.0	9.4	31	57.0	11.9	21
F20	7	11.0	10.4	95	66.6	16.6	25
F21	4	9.7	6.1	63	57.2	17.2	30
range	3-14	9.7-46.0	4.5-22.3	10-95	22.3-87.8	3.5-17.2	4-76

Repeats = number of times the contraction assay was performed on the given strain.

Table S3. Tukey adjusted p-values for all pairwise comparisons of contraction in SF medium between cell strains. High-lighted comparisons are statistically significant ($p < 0.05$).

	F3	F6	F8	F10	F12	F17	F18	F20	F21
F1	0.99	0.99	0.72	0.98	0.99	0.99	0.99	0.02	0.07
F3	---	0.99	0.83	0.99	0.99	0.99	0.99	0.07	0.14
F6	---	---	0.82	0.99	0.99	0.99	0.99	0.09	0.15
F8	---	---	---	0.33	0.69	0.99	0.99	0.94	0.95
F10	---	---	---	---	0.99	0.78	0.80	0.01	0.02
F12	---	---	---	---	---	0.94	0.97	0.08	0.13
F17	---	---	---	---	---	---	0.99	0.87	0.88
F18	---	---	---	---	---	---	---	0.50	0.60
F20	---	---	---	---	---	---	---	---	0.99

Table S4. Tukey adjusted p-values for all pairwise comparisons of contraction in FBS-supplemented medium between cell strains. High-lighted comparisons are statistically significant ($p < 0.05$).

	F3	F6	F8	F10	F12	F17	F18	F20	F21
F1	0.99	0.99	0.99	0.03	< 0.01	0.97	< 0.01	0.06	0.01
F3	---	0.99	0.99	0.05	< 0.01	0.95	0.01	0.11	0.01
F6	---	---	0.99	0.03	< 0.01	0.87	< 0.01	0.07	0.01
F8	---	---	---	0.24	< 0.01	0.99	0.04	0.45	0.07
F10	---	---	---	---	< 0.01	0.89	0.99	0.99	0.99
F12	---	---	---	---	---	< 0.01	0.01	< 0.01	0.01
F17	---	---	---	---	---	---	0.51	0.99	0.58
F18	---	---	---	---	---	---	---	0.91	0.99
F20	---	---	---	---	---	---	---	---	0.95

References for Supplemental Material

1. Carlson MA, Prall AK, Gums JJ: **RNA interference in human foreskin fibroblasts within the three-dimensional collagen matrix**. *Mol Cell Biochem* 2007, **306**(1-2):123-132.
2. Carlson MA, Longaker MT, Thompson JS: **Modulation of FAK, Akt, and p53 by stress release of the fibroblast-populated collagen matrix**. *J Surg Res* 2004, **120**(2):171-177.
3. Nagy R: **Application and Measurement of Ultraviolet Radiation**. *American Industrial Hygiene Association journal* 1964, **25**:274-281.
4. Carlson MA: **Technical note: assay of cell quantity in the fibroblast-populated collagen matrix with a tetrazolium reagent**. *Eur Cell Mater* 2006, **12**:44-48.
5. Grinnell F, Zhu M, Carlson MA, Abrams JM: **Release of Mechanical Tension Triggers Apoptosis of Human Fibroblasts in a Model of Regressing Granulation Tissue**. *Exp Cell Res* 1999, **248**(2):608-619.
6. Sambrook J, Russell DW: **Molecular Cloning: A Laboratory Manual**. Cold Spring Harbor: Cold Spring Harbor Laboratory Press; 2001.
